# A fast likelihood solution to the genetic clustering problem

**DOI:** 10.1111/2041-210X.12968

**Published:** 2018-01-30

**Authors:** Marie‐Pauline Beugin, Thibault Gayet, Dominique Pontier, Sébastien Devillard, Thibaut Jombart

**Affiliations:** ^1^ Univ Lyon Laboratoire de Biométrie et Biologie Evolutive CNRS Université Claude Bernard Lyon 1 Villeurbanne France; ^2^ ANTAGENE, Animal Genomics Laboratory La Tour de Salvagny France; ^3^ Office National de la Chasse et de la Faune Sauvage Unité Cervidés Sangliers Montfort Birieux France; ^4^ Department of Infectious Disease Epidemiology School of Public Health MRC Centre for Outbreak Analysis and Modelling Imperial College London London UK

**Keywords:** EM algorithm, genetic assignment, genetic clustering, hybridisation, microsatellites, population membership, relative performances, SNP

## Abstract

1. The investigation of genetic clusters in natural populations is an ubiquitous problem in a range of fields relying on the analysis of genetic data, such as molecular ecology, conservation biology and microbiology. Typically, genetic clusters are defined as distinct panmictic populations, or parental groups in the context of hybridisation. Two types of methods have been developed for identifying such clusters: model‐based methods, which are usually computer‐intensive but yield results which can be interpreted in the light of an explicit population genetic model, and geometric approaches, which are less interpretable but remarkably faster.
2. Here, we introduce *snapclust*, a fast maximum‐likelihood solution to the genetic clustering problem, which allies the advantages of both model‐based and geometric approaches. Our method relies on maximising the likelihood of a fixed number of panmictic populations, using a combination of geometric approach and fast likelihood optimisation, using the Expectation‐Maximisation (EM) algorithm. It can be used for assigning genotypes to populations and optionally identify various types of hybrids between two parental populations. Several goodness‐of‐fit statistics can also be used to guide the choice of the retained number of clusters.
3. Using extensive simulations, we show that *snapclust* performs comparably to current gold standards for genetic clustering as well as hybrid detection, with some advantages for identifying hybrids after several backcrosses, while being orders of magnitude faster than other model‐based methods. We also illustrate how *snapclust* can be used for identifying the optimal number of clusters, and subsequently assign individuals to various hybrid classes simulated from an empirical microsatellite dataset.
4. *snapclust* is implemented in the package adegenet for the free software R, and is therefore easily integrated into existing pipelines for genetic data analysis. It can be applied to any kind of co‐dominant markers, and can easily be extended to more complex models including, for instance, varying ploidy levels. Given its flexibility and computer‐efficiency, it provides a useful complement to the existing toolbox for the study of genetic diversity in natural populations.

## 1 INTRODUCTION

The identification of groups of genetically related individuals within a population, sensu population subdivision, is an ubiquitous problem in most fields in which genetic data analysis plays an important role including molecular ecology, evolutionary and conservation genetics. Quantifying the magnitude of the population subdivision, assessing whether the genetic differentiation matches with the spatial repartition of subpopulations or not, and, identifying from which genetic units individuals belong or come have been the focus of attention of population geneticist from the inception of population genetics (Wright, 1951). Specific applications include, for example, the definition of panmictic groups (Corander, Waldmann, & Sillanpää, 2003; Falush, Stephens, & Pritchard, 2003; Pritchard, Stephens, & Donnelly, 2000), the classification of isolates into distinct lineages in microbiology (Feil, Li, Aanensen, Hanage, & Spratt, 2004; Maiden et al., 1998), the investigation of social or ecological units in molecular ecology (Jombart, Devillard, & Balloux, 2010; Sugg, Chesser, Stephen Dobson, & Hoogland, 1996), and the identification of various types of hybrids in conservation genetics (Allendorf, Leary, Spruell, & Wenburg, 2001; Anderson & Thompson, 2002; Vähä & Primmer, 2006). Because of this wealth of applications, genetic clustering has received considerable interest from the methodologists community. Seeking the number of genetic clusters from a set of individual genotypes and assigning individuals into clusters has become a gold standard in population genetics, and, a large number of statistical methods have been developed and used routinely for nearly two decades (Anderson & Thompson, 2002; Corander et al., 2003; Falush et al., 2003; Jombart et al., 2010; Pritchard et al., 2000).

While there is no single taxonomy of methods, a natural separation can be made between “model‐based” approaches, which use a population genetics model to compute a likelihood, including maximum‐likelihood (ML) and Bayesian methods (Anderson & Thompson, 2002; Corander et al., 2003; Dupanloup, Schneider, & Excoffier, 2002; Falush et al., 2003; Pritchard et al., 2000), and “geometric” approaches, which cluster individuals based on their distances in the genetic space spanned by allelic data, without assuming a specific population genetics model (Feil et al., 2004; Jombart et al., 2010). In genetic clustering problems, the likelihood is defined as the probability that the set of genotypes under consideration was generated under a given population structure and model of evolution. As such, these methods are more readily interpretable: individual group membership probabilities genuinely reflect the probability that the individual “belongs” to the different groups. Unfortunately, these methods are typically computer‐intensive, as they involve the exploration of a high‐dimensional parameter space, using optimisation procedures (Dupanloup et al., 2002) or Markov Chain Monte Carlo (MCMC) techniques (Corander et al., 2003; Falush et al., 2003; Pritchard et al., 2000; Vähä & Primmer, 2006). While more efficient implementations have been developed (Alexander, Novembre, & Lange, 2009; Raj, Stephens, & Pritchard, 2014; Tang, Peng, Wang, & Risch, 2005), geometric approaches remain an appealing alternative, as they are typically orders of magnitude faster, while producing comparably accurate results under a range of simulation scenarios (Jombart et al., 2010). The main limitation of geometric approaches lies in the fact that their results are harder to interpret biologically. Indeed, these methods typically identify clusters from pairwise genetic distances, without providing group membership probabilities (Jombart et al., 2010; Legendre & Legendre, 2012), so that weak separation between clusters or admixture patterns cannot be distinguished from strong, clear‐cut population structure. To some extent, this issue can be addressed, using exploratory approaches such as the DAPC (Jombart et al., 2010), to visualise cluster diversity in a reduced space and even estimate group assignment probabilities, but these probabilities merely reflect genetic proximities, and cannot be interpreted as probabilities that an individual belongs to a given population.

Here, we combine both types of approaches to formulate a new clustering method called “*snapclust*,” which retains the advantages of both worlds. Our method relies on the most common population genetics model which underlies the Hardy–Weinberg (HW) equilibrium to compute the likelihood of a given clustering solution. Rapid convergence to ML estimates of clusters is achieved by combining geometric approaches (Jombart et al., 2010; Legendre & Legendre, 2012) and the Expectation‐Maximisation (EM) algorithm (Dempster, Laird, & Rubin, 1977). In practice, our method allows to select the optimal number of clusters within a set of genotypes, and provides results where group assignment scores are genuine probabilities that a given genotype was generated in various populations under HW model, while remaining essentially as fast as geometric approaches (Jombart et al., 2010). Our method can also be used for identifying various types of hybrids between two parental populations. Besides, being an ML estimation method, *snapclust* can also be combined with goodness‐of‐fit statistics such as Akaike information criterion (AIC; Akaike, 1998) or the Bayesian information criterion (BIC; Schwarz, 1978) to guide the choice of the optimal numbers of clusters.

In this paper, we describe the model underlying *snapclust* and its implementation, and then compare the performance of our method with current gold‐standards for genetic clustering (STRUCTURE; Pritchard et al., 2000; Falush et al., 2003), BAPS, adegenet's *find.cluster* (Jombart et al., 2010) and hybrid identification (NEWHYBRIDS; Anderson & Thompson, 2002). Using a large number of simulations, we assessed the impact of the number of loci, the dispersal model, the level of genetic differentiation between populations, and the number of populations (when looking at multiple clusters without hybrids), on the performance of the different methods. We also provide a worked example based on the analysis of a simulated dataset to illustrate typical results provided by the method. Here, *snapclust* is implemented in the package adegenet (Jombart, 2008; Jombart & Ahmed, 2011) for the R software (R Core Team 2017), thus being readily compatible with a wealth of tools for genetic data analysis in R (Goudet, 2005; Jombart et al., 2017; Kamvar, Tabima, & Grünwald, 2014; Paradis, 2010; Popescu, Huber, & Paradis, 2012).

## 2 MATERIALS AND METHODS

### 2.1 Rationale of *snapclust*

#### 2.1.1 Model likelihood

We consider a dataset of allelic profiles *x *= {*x*_*i,j*_} where *i* indexes individuals (*i *=* *1, …, *N*) and *j* indexes loci (*j *=* *1, …, *J*), so that ***x***_***i,j***_ is a vector of allele counts for individual *i* at locus *j*. The likelihood of our model is defined as the probability of observing these data given a clustering solution ***g ***= {*g*(*i*)}, where *g*(*i*) defines the group of individual *i*, with groups indexed by *k *=* *1, …, *K*. Under the HW model, this likelihood is defined as:

$$p\left(x_{i,j}|f_{g(i),j},\;\Pi \right)=M\left(x_{i,j},\;f_{g(i),j},\;\pi \right)$$

where *M* is the probability mass function of the multinomial distribution, ***f***_***g*****(*****i*****),*****j***_ is the vector of allele frequencies in group *g*(*i*) at locus *j*, and π is the ploidy of the organism considered. Allele frequencies within a group are directly computed as the relative frequencies of each allele in this group. Assuming independence between loci, the likelihood term for the genotype *i* is given by the following:

$$p\left(x_{i}|f_{g(i)},\;\pi \right)=\Pi_{j}p\left(x_{i,j}|f_{g(i),j},\;\pi \right)$$

where ***f***_***g*****(*****i*****)**_ = {*f*_*g*(*i*),1_ ,…, *f*_*g*(*i*),*J*_} and ***x***_***i***_ = {*x*_*i*,1_, …, *x*_*i*,*J*_}. If we further assume independence of individuals conditional on their group memberships, the general likelihood is given by the following:

$$p\left(x|f,\;g,\pi \right)=\Pi_{i}p\left(x_{i}|f_{g(i)},\;\pi \right)$$

where *f* = {*f*_*1* _,…, *f*_*K*_}. In practice, we will consider the log‐likelihood of a clustering solution defined as follows:

$$LL\left(g\right)=\sum_{i}\sum_{j}M\left(x_{i,j},\;f_{g(i),j},\pi \right)$$

Note that while the current implementation of *snapclust* considers a constant ploidy across individuals and loci, the formula above can readily be extended to varying ploidy, in which case π will become an individual‐ or locus‐specific term.

Assuming that all clusters have been sampled, the probability *p*(*g*(*i*) = *k*) that an individual *i* belongs to a group *k* is defined by the standardised likelihood:

$$p\left(g\left(i\right)=k\right)=p\left(x_{i}|g(i)=k,\;f_{k},\;\pi \right)/\sum_{q}p\left(x_{i}|g\left(i\right)=q,\;f_{q},\;\pi \right)$$

#### 2.1.2 Modelling hybridisation

The clustering model above can be readily extended to accommodate the presence of hybrids. For simplicity, we consider a case where hybrids are obtained from two parental populations *A* and *B*. The allelic composition *f*_*H,j*_(*w*) of a hybrid population *H* at locus *j* is defined as a mixture of the allele frequencies of two parental populations, *f*_*A,j*_ and *f*_*B,j*_. This mixture is defined by the hybridisation coefficient *w*, which indicates the proportion of the genomes of the hybrid population coming from the parental population *A*, so that:

$$f_{H,j}\left(w\right)=wf_{A,j}+\left(1-w\right)f_{B,j}$$

Modelling of hybridisation through the coefficient *w* is very flexible, as it enables the specification of any kind of hybrids between *A* and *B*. For instance, first‐generation hybrids (F1) correspond to *w *=* *0.5, while first‐ and second‐generations backcrosses with A that correspond to *w *=* *0.25 and *w *=* *0.125 respectively. The likelihood of a hybrid is defined as before, but using the allele frequencies mixture as follows:

$$p\left(x_{i}|g(i)=H,\;f_{A},\;f_{B},\;w,\;\pi \right)=\Pi_{j}p\left(x_{i,j}|f_{H,j},\;\pi \right)$$

#### 2.1.3 Optimisation procedure

Here*, snapclust* achieves fast likelihood maximisation using the EM algorithm (Dempster et al., 1977), in which the vector of group membership *g* is treated as a latent variable. In this respect, our approach is closely related to *K*‐means clustering, except that *snapclust* maximises a log‐likelihood rather than between‐group distances (Jombart et al., 2010). The EM algorithm proceeds by alternating computation of the likelihood, and assignment of individuals to their most likely cluster. Allele frequencies are updated at each iteration, using their maximum likelihood estimation, that is, the mean frequencies of alleles in individuals of a given group. The algorithm, adapted from the use of EM for maximising likelihood in mixed distribution problems (Fraley & Raftery, 2002), can be formalised through the following steps:

1. define initial group assignments *g* (see “starting point” below)
2. (*expectation* step) update allele frequencies *f* within each group, computed as the relative frequencies of alleles amongst individuals of this group; compute group membership probabilities *p*(*g*(*i*) = *k*) for all individuals *i* and groups *k*
3. (*maximisation* step) update the group definition *g*: based on group membership probabilities computed in step 2, assign each individual to their most likely group
4. return to step 2 until convergence

We assume convergence when the difference in log‐likelihoods in two successive iterations becomes negligible, that is, is less than an arbitrary threshold (set to 10^−10^ by default).

#### 2.1.4 Starting point

The EM algorithm typically converges very fast, generally within 10 iterations in the simulated and empirical datasets described here. Unlike some other optimisation procedures and MCMC, it is a deterministic algorithm, so that it always converges to the same solution for a given starting point (step 1). As a consequence, it is unfortunately also prone to being trapped in local maxima, yielding suboptimal results for some starting points. To avoid this issue, we implemented several options to define the initial clusters used as starting point of the algorithm. The first strategy, borrowed from the original implementation of *K*‐means in R (R Core Team, 2017), is a “brute force” approach in which the algorithm is run multiple times, using each time a randomly defined group assignment, and retaining the solution with the highest likelihood. The second strategy which we introduce here is to use fast geometric approaches such as Ward's clustering (Legendre & Legendre, 2012) or *K*‐means after dimension reduction (Jombart et al., 2010) to set up the initial clusters. Based on our simulated datasets, random initial groups with 50 independent replicates, *K*‐means, and Ward initialisation all gave similar results. By default, we recommend using Ward as it will be faster for most datasets. The three methods are available in the implementation of the algorithm, as well as any other user‐defined initial clusters.

#### 2.1.5 Finding the optimal number of clusters

The advantage of using a ML approach is that different models can be compared using classical goodness‐of‐fit statistics. While a full comparison of model selection techniques for genetic clustering is beyond the scope of the present paper, we have implemented four different information criteria shown to be useful for selecting the true number of clusters in the case of mixtures of distributions (Akogul & Erisoglu, 2016). These statistics all rely on measuring the lack of fit of the model (deviance), and use different penalties for the complexity of the model (number of free parameters). The first, AIC (Akaike, 1998), is probably the most frequently used for models comparison. Noting *L*’ the estimated maxima of *LL*(*g*), the AIC of our model is computed as:

$$\text{AIC}=-2L^{\prime }+2\left(K\left(P-J\right)\right)$$

where the first term is the deviance of the model, and the second term corresponds to the complexity of the model, with *P* being the total number of alleles in the dataset across *J* loci. The complexity reflects the fact that for each of the *K* groups, (*P* − *J*) independent allele frequencies are estimated, so that the total number of free parameters of the model is (*K* (*P* − *J*)). We also implemented the variant of the AIC for small sample sizes, defined as (Akogul & Erisoglu, 2016):

$${\text{AIC}}_{c}=-2L^{\prime }+2\left(K\left(P-J\right)N\right)/\left(N-KP+KJ-1\right)))$$

A popular alternative to AIC and AICc is the BIC (Schwarz, 1978), which also relies on a penalised deviance, albeit putting a stronger cost on complexity:

$$\text{BIC}=-2L^{\prime }\;+\;ln\left(N\right)\left(K\left(P-J\right)\right)$$

Finally, we also implemented the Kullback Information Criterion (KIC, Cavanaugh, 1999), which gave the best overall results for detecting the number of clusters from mixtures of multivariate normal distributions (Akogul & Erisoglu, 2016):

$$\text{KIC}=-2L^{\prime }+3\left(K\left(P-J\right)+1\right)$$

All these statistics have similar behaviours in that the lower values typically indicate better fits. In practice, a sharp decrease in the statistics values with increasing numbers of clusters is most likely to reveal the optimal numbers of clusters (Jombart et al., 2010).

#### 2.1.6 Implementation and availability

*snapclust* is implemented in the R package adegenet (Jombart, 2008; Jombart & Ahmed, 2011) version 2.1.0, available via R's native package installation system as well as on github (https://github.com/thibautjombart/adegenet). The function snapclust.em implements the basic method, including different options for defining the initial state of the EM algorithm and the model for hybrids classification. The functions AIC, AICc, BIC and KIC implement the respective goodness‐of‐fit statistics. The function snapclust.em.choose.k derives clustering solutions for increasing numbers of clusters and computes the associated goodness‐of‐fit statistics, so that it can guide the choice of the optimal number of clusters. The method is documented in a dedicated online tutorial available by typing adegenet::adegenetTutorial(‘snapclust’) in a R session. Code and documentation are released under GPL ≥ 2 license.

### 2.2 Simulations

#### 2.2.1 Simulated datasets without hybrids

The datasets were simulated using quantiNEMO (Neuenschwander, Hospital, Guillaume, & Goudet, 2008) with the parameters indicated in Table 1. We chose to simulate single nucleotide polymorphism (SNP) markers and explored a wide range of possible configurations by varying four simulation parameters: the number of loci, the dispersal model, the rate of dispersion, and the number of populations. The different rates of dispersal led to different levels of differentiation between populations. All combinations of dispersal rate and number of loci were tested as the number of loci and the differentiation level are expected to define jointly the resolution of a panel of genetic markers (Vähä & Primmer, 2006). This led to 36 combinations of parameters. Ten independent random replicates were obtained for each combination leading to 360 simulated datasets. To avoid prohibitive computational times, we allowed the number of populations and the dispersal model to vary randomly across replicate, rather than adding systematically new combinations of parameters to the pool of data to simulate. The number of individuals per population was fixed to 100.

**Table 1:** Parameters used in the simulations using the computer program quantiNEMO

Parameter	Value	Parameter	Value
Generations	10,000	Population size	100
Number of loci	[20, 50, 80, 150, 300, 500]	Number of alleles	2
Dispersal model	Migrant‐pool island model or 1‐D stepping stones model	Mutational model	K‐allele model
Dispersal rate	[0, 0.001, 0.002, 0.003, 0.005, 0.01]	Mutation rate	0
Number of populations	2–15	Mating system	Random mating

#### 2.2.2 Simulated datasets with hybrids

The simulated datasets used for the clustering of hybrids were derived from the previous simulations, by sampling two parental populations (P1, P2) at random in each of the 360 simulated datasets described before. For each, hybrids were simulated using the function hybridize of the adegenet package to obtain F1 hybrids (P1 × P2), first‐generation backcrosses (BC1: F1 × P1 and F1 × P2), and second‐generation backcrosses (BC2: (F1 × P1) × P1 and (F1 × P2) × P2). Each simulated dataset was formed by 100 individuals from P1 and P2 each, and 10 individuals from each hybrid class (i.e. 50 hybrids in total). While arbitrary, these sample sizes yielded a sufficient number of hybrids to analyse while retaining enough individuals to characterize the genetic makeup of parental populations.

#### 2.2.3 Analyses of simulated datasets without hybrids

Our simulation study focussed on comparing *snapclust* to existing standard for the assignment of individual genotypes to groups (rather than inferring the true number of clusters). Therefore, the number of clusters was fixed to the known number of populations within the simulated dataset for all presented analyses. The clustering of individuals in absence of hybrids was performed using the *snapclust*, structure 2.3.4 (Falush et al., 2003; Hubisz, Falush, Stephens, & Pritchard, 2009; Pritchard et al., 2000), BAPS 5.4 (Cheng, Connor, Sirén, Aanensen, & Corander, 2013; Corander et al., 2003; Tang, Hanage, Fraser, & Corander, 2009), and adegenet's find.clusters (Jombart et al., 2010). The *snapclust* analysis was carried out using default parameters (group assignment initialisation using the “ward” option). STRUCTURE analyses were carried out, using an admixture model with correlated allele frequencies between populations and no *a priori* information on population membership. The program was run ten times for result consistency purposes, with MCMC length of 500,000 after a burn‐in of 100,000 iterations. Individuals were assigned to the cluster for which their posterior assignment probability was the highest. For BAPS, we performed a “mixture clustering” analysis. Finally, we ran the function find.clusters retaining 90% of the total variation in the initial dimension reduction step.

As clusters identified in these previous analyses are not labelled, it was impossible to judge if individuals were assigned to their true cluster. To assess the quality of the results and compare the different methods, we used pairwise comparisons of individuals instead, examining whether pairs of individuals where adequately placed in the same, or different clusters. We used two complementary measures to do so calculated on each of the 360 simulated dataset analysed. The *true positive rate* (*TPR*) was defined as the proportion of individuals belonging to the same population which were indeed clustered together by the method. The *true negative rate* (*TNR*) was defined as the proportion of individuals which did not belong to the same population and were adequately placed in different groups by the method. Note that the Rand index (Rand, 1971), which can be used for comparing unlabelled clusters, is proportional to (*TPR* + *TNR*), so that the present analyses should give a more detailed account of clustering results than the Rand index alone. The impact of the different simulation parameters on *TPR* and *TNR* was assessed using separate multivariate linear regressions. As classical linear regression is designed to predict a response variable which can take any positive or negative values, a logit transformation was applied to the proportions, so that log(*TPR*/1 − *TPR*) and log(*TNR*/1 − *TNR*) were used as response variables. We preferred a linear regression on these rates to a binomial generalized linear model (GLM) for practical reasons. The calculation of *TPR* and *TNR* being based on all pairwise comparisons of individuals within simulated datasets, a binomial GLM would have required millions of observations to be included, leading to prohibitive computations. Instead, 360 TPR and TNR values were analysed, that is, one per simulation.

We tested for the effects of the number of loci, the dispersal model, the overall *F*_st_ between simulated populations, the number of populations, and the clustering method. We also investigated potential two‐way interactions between the clustering method and the four simulation parameters we varied (Table 1), as well as between the number of loci and the *F*_st_. Backward stepwise model selection based on AIC was used to retain significant predictors, and confirmed using classical likelihood ratio tests. Bonferroni correction was used to account for multiple testing with a target type 1 error of 1%. When assessing the overall differences between methods across all simulations and thus across all conditions of differentiation, number of loci and populations and dispersal model, we compared *TPR* and *TNR* predicted by the respective models by transforming predicted logit rates back to their original scale.

#### 2.2.4 Analyses of simulated datasets with hybrids

The clustering of individuals in the presence of hybrids was carried out using *snapclust* and the computer program newhybrids (Anderson & Thompson, 2002). The *snapclust* analysis was carried out, using the default parameters and specifying hybridisation coefficients for F1, first (BC1) and second (BC2)‐generation backcrosses (hybrid.coef values: 0.5, 0.25, 0.125). The NEWHYBRIDS analysis was carried out, using Jeffreys's prior and setting the burn‐in period to 100,000, with a MCMC length of 500,000 iterations. Ten repetitions were carried out for each simulated dataset. Unlike the previous comparison, parental and hybrid classes are labelled, so that it was possible to compare the methods by directly examining how well they assigned individuals to their actual hybrid group, using the *mean correct group assignment*, computed as the proportion of individuals whose type (parental, F1, BC1 and BC2) was correctly identified. In addition, we also examined the group membership probability calculated by each method for the true group, later referred to as the “*support*” for the true group. As before, the impact of the different simulation parameters on the performance of the methods was assessed, using multiple linear regression on logit probabilities, with separate models for the mean correct group assignment, and the support to the true group. In both cases, the following predictors were included: number of loci, dispersal model, *F*_st_, as well as the hybrid class (parental, F1, first or second back‐cross), and the clustering method. Interaction were investigated between the method and the simulation parameters, and between the number of loci and the *F*_st_. As before, variable selection was achieved using backward stepwise selection based on AIC and likelihood ratio tests, using a Bonferroni correction to account for multiple testing with a target type 1 error of 1%.

### 2.3 Illustration using microsatellite data

To complement the simulation study which assessed the overall performances of our method, we illustrated its practical application by reproducing a typical analysis of microsatellite markers data, starting with the identification of the most likely number of clusters, followed by the assignment of individuals to groups, and the description of relationships between groups. We simulated hybrids from an empirical dataset of 30 microsatellite markers typed for 15 breeds (Laloë, Jombart, Dufour, & Moazami‐Goudarzi, 2007), distributed as the “microbov” dataset in adegenet. Parental populations were obtained by sampling 30 individuals from the Lagunaire and 30 from the Salers populations. Hybrids were simulated using the function hybridize, to obtain 30 F1 hybrids, and then 30 of each first and second backcrosses, resulting in 210 individuals. While arbitrary, these numbers replicate a situation where hybrids are more numerous than parental populations, as could be the case in nature when studying large hybridisation zones.

We first carried out a global clustering analysis on this dataset, looking for the optimal number of clusters, using AIC (function snapclust.em.choose.k) in order to confirm that *K* = 2 parental populations was the optimal solution. We then looked for potential hybrids (function snapclust.em), using hybridisation coefficients corresponding to F1 (0.5), first‐generation backcross (0.25, 0.75) and second‐generation backcross individuals (0.125, 0.875). Group membership probabilities were visualised using the function compoplot. As a complement, we also explored the diversity between hybrid classes using a discriminant analysis of principal components (DAPC; Jombart et al., 2010), employing cross‐validation to determine the optimal number of principal components to retain. The R script required to reproduce the simulated data and run the analyses are provided in Supplementary Material.

## 3 RESULTS

### 3.1 Clustering of individuals without hybrids

**Figure 1:**
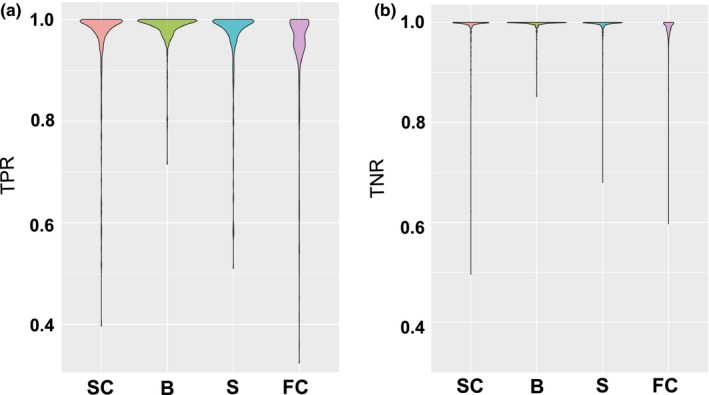
Comparison of the various methods on simulated genetic clusters. *Notes*: This figure shows the distribution of (a) the true positive rates (*TPR*) and (b) true negative rates (*TNR*) obtained over all the 360 simulations for the four different methods: *snapclust* (SC), BAPS (B), STRUCTURE (S) and *find.clusters* (FC) for the clustering of individuals in absence of hybrids. This width of the enveloppes reflects the density of points

All four different methods exhibited very good performances in terms of *TPR* (most results above 90%) and near perfect *TNR*, showing that clusters present in the simulated dataset were overall well recovered by all approaches (Figure 1). Runtime analysis showed that *snapclust* was on average 27 times faster than BAPS and about 120,000 times faster than STRUCTURE, with an average analysis time below a second (Table S1).

Multivariate linear regression captured a large fraction of the variation in logit(*TPR*) values (Adjusted *R* ^2^: 61%, Table S2). Predicted *TPR* and *TNR* we pooled by methods across all simulations to compare overall performances of the different approaches. The results showed marginal variations in performances of the methods, with mean predicted *TPR* varying from 96.7% (IQR: 95.4%–99.1%) for *find.clusters* to 99.0% (IQR: 98.5%–99.7%) for BAPS, with 98.0% (IQR: 97.1%–99.5%) for STRUCTURE and 98.1% (IQR:97.3%–99.6%) for *snapclust*. Similar results were observed for *TNR* values, although the model explained a smaller fraction of the variance (Adjusted *R* ^2^: 44%, Table S3). Predicted *TNR* values were very high for all four methods: 96.7% (IQR: 95.4%–99.1%) for *find*.clusters, 98.9% (IQR: 98.6%–99.7%) for BAPS, 98.1% (IQR: 97.1%–99.5%) for STRUCTURE, and 98.1% for *snapclust* (IQR: 97.3%–99.5%).

Other parameters impacted the performances of the different methods in terms of *TPR* and *TNR* (Tables S2 and S3). In both cases, increased number of loci and larger *F*_st_ generally improved *TPR* and *TNR* values, although a saturation effect was observed so that the effects of large numbers of loci and stronger *F*_st_ effectively cancelled out. For instance, for *F*_st_ of approximately 0.1, the increase in the number of loci from 50 to 500 allowed to increase the *TPR* from 0.88 to 0.97 across all methods, while the same increase in the number of loci for *F*_st_ of approximately 0.6 yielded *TPR* values ranging between 0.99 and 1. In addition, increasing the number of populations led to improved *TNR* (Table S3).

### 3.2 Clustering of individuals with hybrids

**Figure 2:**
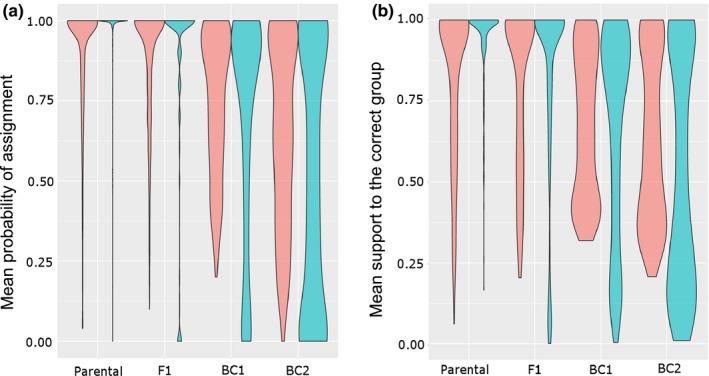
Comparison of *snapclust* (red) and NEWHYBRIDS (blue) for the identification of hybrids using simulated data(. *Notes*: This figure shows the distributions of (a) the mean proportion of correct group assignment and (b) the support (i.e. group membership probability) for the true class across all simulated datasets. Three hybrid classes are considered in the simulations in addition to the parental class: first‐generation hybrids (F1), first‐generation backcrosses (BC1) and second‐generation backcrosses (BC2). This width of the enveloppes reflects the density of points

Results based on the proportion of correct assignment and the support to the true group both showed similar patterns, with stark contrast between *snapclust* and NEWHYBRIDS (Figure 2, Tables S4–S5). The final model of the proportion of correct assignment explained most of the variation in the results (adjusted *R* ^2^ = 63.78%). Increased number of loci (*t *=* *23.32; *p* = 2.74 × 10^−110^) and stronger *F*_st_ (*t *=* *31.28; *p* = 5.094 × 10^−185^) generally improved group prediction, although a significant yet negligible saturation effect was observed between the two (*t *=* *−5.046; *p* = 4.78 × 10^−7^). While hybrid classes were on average harder to identify than parental populations, with the lowest success observed for deeper backcrosses, the two methods behaved very differently: NEWHYBRIDS seemed to recover parental populations more efficiently, but *snapclust* exhibited improved performances for the identification of hybrids in deeper levels of hybridisation (Figure 2, Table S4–S5). This contrast was strongest for BC2, in which the odd ratio of accurate group predictions averaged to 4.80 in *snapclust* (95% CI: 2.09–11.01) compared to NEWHYBRIDS. Results were qualitatively identical when examining the support to the true group (Figure 2, Table S5), although the difference in odd ratio for BC2 was smaller, with an average of 1.74 (95% CI: 1.05–2.87). As for the clustering comparison, *snapclust* also proved more computer efficient, being on average 525,000 times faster than NEWHYBRIDS, with an average runtime of 0.54 s.

### 3.3 Illustration on the microbov data

**Figure 3:**
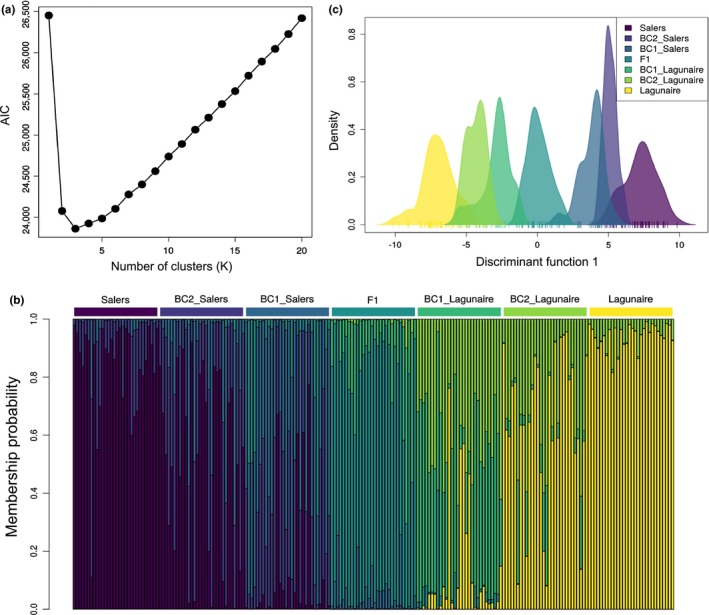
Illustration of *snapclust* using simulated hybrids from cattle breed microsatellite data. *Notes*: (a) Representation of the Akaike Criterion value according to the number of populations (*K*) considered. (b) Representation of the individual probability of assignment obtained with the function *snapclust.em* for the different types of individuals present in the dataset. (c) Representation of the first axis of the discriminant analysis of principal components carried out on the hybrid groups found using the *snapclust* analysis

AIC values computed for increasing values of *K* showed a sharp decrease at *K* = 2, with only marginal improvements for *K* = 3, hinting to the existence of two major clusters (Figure 3a) here formed by the parental populations (Salers and Lagunaire). Other goodness‐of‐fit statistics (AICc, BIC, KIC) also pointed to *K* = 2, but AIC showed the most clear‐cut result (Figure S1). Subsequent analysis with *snapclust* including F1 hybrids as well as first‐ and second‐generation backcrosses shows well‐identified parental clusters, as well as a large number of individuals assigned to the hybrid classes (Figure 3b). Parental and F1 hybrids groups were well identified, with 98.3% and 93.3% of successful individual assignment, respectively. Deeper hybrid classes were much harder to recover, with 51.7% of the BC1 and only 16.7% of BC2 correctly identified. This result is, however, in line with expectations in the presence of weak genetic differentiation. Indeed, while moderate genetic differentiation was observed between parental populations (*F*_st_ = 0.157), the average differentiation between BC2 and the “neighbouring” groups (closest parent and BC1) was negligible (*F*_st_ < 0.01). This lack of differentiation was confirmed by a DAPC retaining 20 dimensions (Figure S2), which showed that individuals were structured along a cline of genetic differentiation between the two parental populations, with considerable overlap between “neighbouring” groups (Figure 3c).

## 4 DISCUSSION

We have introduced “*snapclust*,” a new genetic clustering method which achieves fast maximum likelihood identification of the optimal number of clusters within a set of genotypes, assignment of individuals to panmictic populations, and can also be used to detect various classes of hybrids. The analyses of simulated data show that our method performs as well as current gold standards for genetic clustering under the investigated models. Indeed, while statistically significant differences were observed in *TPR* and *TNR* across methods with BAPS exhibiting the best results, these differences were small in terms of absolute performance: predicted *TPR* was 97% for *snapclust* compared to 98% for BAPS, and predicted *TNR* exceeded 99% for both methods. When used to detect hybrids, *snapclust* exhibited different performances from NEWHYBRIDS, being less accurate for identifying parental populations but better at recovering deeper hybrid classes such as second‐generation backcrosses, while being again tremendously more computer efficient. The combination of likelihood estimation and EM algorithm for cluster detection is not new (Fraley & Raftery, 2002), and has been used successfully as a fast yet powerful alternative to more complex likelihood‐based methods in other fields than population genetics (Fraley & Raftery, 1998). As such, we believe the kind of approach introduced here offers exciting prospects for extending previous efforts to make model‐based genetic clustering methods more computer‐efficient (Alexander et al., 2009; Raj et al., 2014; Tang et al., 2005).

The fact that *snapclust* is orders of magnitude faster than other model‐based approaches gives it a substantial practical advantage, especially when the analysis needs to be run multiple times, as is the case when investigating different values of *K*, when conducting a simple simulation study, or when using resampling strategy to assess statistical uncertainty. This latter aspect in particular is worth investigating, as our method does not, unlike Bayesian approaches (e.g. BAPS, STRUCTURE) include a natural measure of uncertainty in the form of distributions of group membership probabilities for each individual. For *snapclust*, an alternative approach to assess statistical uncertainty may be to use bootstrap, in which case the method would be to run a large number of times (e.g. 100) on datasets obtained by random resampling (with replacement) of the loci. Such approach would provide a distribution of group membership probabilities for each individual (one per run), and thereby a measure of uncertainty. Bootstrap on loci can readily be implemented, using existing tools for genetic data handling (Jombart, 2008; Jombart & Ahmed, 2011; Kamvar et al., 2014). It would be relatively easy to apply in the case of hybridisation between two parental clusters, in which case clusters are labelled, and therefore comparable across different runs. In the general case of unlabelled clusters, however, the difficulty of matching clusters across different runs will first need to be overcome for this approach to be applied.

While our simulation study required substantial computational resources, there are undoubtedly many more scenarios and methods to explore, involving a wider range of population genetics models, optimisation procedures, and potentially various types of genetic markers. The relative effects of selection, recombination, and linkage disequilibrium remain to be investigated. The latter may be of first concern, as it would break the assumption of independence between loci, in which case our model only approximates the actual, unknown likelihood. This said, the very same assumption underpins maximum likelihood phylogenetic reconstruction, which has nonetheless proved tremendously useful over the past decades (Felsenstein, 1981, 2004). We also note that our simulation study compared assignment of individuals to groups across different methods, assuming the true number of clusters was known. Examining performances for inferring the optimal number of clusters would have led to prohibitive computational times, and was beyond the scope of this study. Further work dedicated to investigating this specific issue would undoubtedly be useful. In particular, the choice of the adequate measure of goodness‐of‐fit, and the potential impact of maximum likelihood approximation through the EM algorithm should be given further consideration. With this in mind, we implemented four different statistics measuring the goodness‐of‐fit of clustering solutions, which should hopefully provide the needed flexibility for future investigations of the “true *K*.”

In our simulations, the number of loci and levels of genetic differentiation varied independently, so that the *resolution* of the datasets may not have been sufficient for detecting some of the hybrid classes, especially the second‐generation backcrosses (Vähä & Primmer, 2006). While this was not a problem for comparing the relative performances of *snapclust* and NEWHYBRIDS, ensuring sufficient resolution should be a primary concern in empirical studies. Ideally, further work will formulate guidelines for defining the minimum resolution required for recovering specific hybrid classes. As a pragmatic alternative, we suggest comparing clustering solutions involving different degrees of hybridisation, and selecting the model providing the best fit of the data (e.g. sensu AIC).

The approach described here is flexible, as it can accommodate any type of co‐dominant markers including microsatellites and SNPs, and can readily be extended to varying ploidy levels. Interestingly, it can also be extended to other genetic models as well, including potentially more complex ones. Contrary to Bayesian approaches which need hundreds of thousands or even millions of iterations to reach mixing and provide a representative sample from the posterior distribution, our fast likelihood maximisation using the EM algorithm converges in a few iterations—typically less than 10 in our simulations. As a consequence, our approach could have great potential for addressing more complex population genetics model, as long as their likelihood is tractable or can be reasonably approximated. One potential obstacle to such extensions is that group memberships need to be treated as a discrete variable, where individuals essentially belong to one group. This will exclude mixture models in which individuals effectively have multiple origins. A workaround for this issue may be to model “mixed groups” explicitly, as we have done in our hybridisation model.

Our method is implemented in the R package adegenet (Jombart, 2008; Jombart & Ahmed, 2011), which supports a wide range of data including microsatellites, SNPs, and amino‐acid sequences, and implements several methods for exploring genetic data (Jombart, Pontier, & Dufour, 2009), revealing spatial patterns (Jombart, Devillard, Dufour, & Pontier, 2008), or investigating genetic clusters (Jombart et al., 2010). Interoperability between different tools has been a long‐standing issue in genetic data analysis (Excoffier & Heckel, 2006). We hope the availability of *snapclust* in the same environment as a wealth of other tools for population genetics (Archer, Adams, & Schneiders, 2017; Goudet, 2005; Kamvar et al., 2014; Paradis, 2010) and phylogenetics (Bortolussi, Durand, Blum, & François, 2006; Jombart et al., 2017; Popescu et al., 2012; Revell, 2012; Schliep, 2011) will enhance its usefulness for the community.

## DATA ACCESSIBILITY

This work does not involve empirical datasets. The dataset microbov, used in our illustration, is distributed with the R package adegenet: https://cran.r-project.org/web/packages/adegenet/index.html
