# Supplementary material for: A fast likelihood solution to the genetic clustering problem
Source: Methods Ecol Evol. 2018 Jan 30;9(4):1006–16. doi: 10.1111/2041-210X.12968 (PMC5993310; doi:10.1111/2041-210X.12968)
Supplement: Supplementary file 1 [file MEE3-9-1006-s001.docx]

**Table S1: runtimes of the different methods for the clustering and hybridisation analyses.** Values indicate the median time computed across all simulations, with the 95% percentile interval indicated between square brackets. Computing times indicated for Structure and Newhybrids correspond to the computing time for one repetition. Time is indicated either in seconds (‘s’) or in hours (‘h’).

|  | **Clustering without hybrids** | **Clustering with hybrids** |
| --- | --- | --- |
| **snapclust** | 0.89s [0.11s ; 6.45s] | 0.54s [0.16s ; 2.27s] |
| **BAPS** | 24s [7s ; 89.05s] | NA |
| **STRUCTURE** | 29.36h [2.17h ; 377.25h] | NA |
| **find.clusters** | 0.15s [0.02s ; 3.09s] | NA |
| **Newhybrids** | NA | 78.87h [10.15h ; 503.24h] |

**Table S2: effect of different variables on the simulation results in the absence of hybrids on the True Positive Rate (*TPR*).** This table provides the summary of the final multivariate regression carried out on logit(*TPR*). Bold font indicates significant results after Bonferroni correction with a target type 1 error of 1%. Contrasts were set for ‘Method’ with STRUCTURE as the intercept.

|  | **Estimate** | **Std. Error** | **t value** | **Pr(>\|t\|)** |
| --- | --- | --- | --- | --- |
| **Intercept** | **1.94** | **0.18** | **10.74** | **6.54e-26** |
| **Number of loci** | **0.0043** | **0.00040** | **9.93** | **1.68e-22** |
| Number of populations (K) | -0.038 | 0.016 | -2.44 | 0.015 |
| **Fst** | **6.61** | **0.26** | **25.16** | **7.74e-116** |
| Method_snapclust | 0.023 | 0.25 | 0.094 | 0.93 |
| **Method_BAPS** | **1.0045** | **0.25** | **4.095** | **4.46e-05** |
| **Method_find.clusters** | **0.89** | **0.25** | **3.62** | **0.00031** |
| **Number of loci : Fst** | **-0.0058** | **0.00070** | **-8.47** | **5.88e-17** |
| Fst : Method_snapclust | -0.036 | 0.33 | -0.11 | 0.91 |
| Fst : Method_BAPS | -0.95 | 0.33 | -2.91 | 0.0037 |
| **Fst : Method_find.clusters** | **-1.43** | **0.33** | **-4.35** | **1.43e-05** |
| Nb_Loci : Method_snapclust | -0.00010 | 0.00050 | -0.11 | 0.91 |
| Nb_Loci : Method_BAPS | -0.0014 | 0.00050 | -2.88 | 0.0041 |
| Nb_Loci : Method_find.clusters | -0.00020 | 0.00050 | -0.32 | 0.75 |
| Nb_populations : Method_snapclust | 0.0074 | 0.022 | 0.34 | 0.74 |
| Nb_populations : Method_BAPS | 0.0085 | 0.022 | 0.39 | 0.70 |
| **Nb_populations :Method_find.clusters** | **-0.12** | **0.022** | **-5.35** | **9.98e-08** |

**Table S3: effect of different variables on the simulation results in the absence of hybrids on the True Negative Rate (*TNR*).** This table provides the summary of the final multivariate regression carried out on logit(*TNR*). Bold font indicates significant results after Bonferroni correction with a target type 1 error of 1%. Contrasts were set for ‘Method’ with STRUCTURE as the intercept.

|  | **Estimate** | **Std. Error** | **t value** | **Pr(>\|t\|)** |
| --- | --- | --- | --- | --- |
| Intercept | 3.59 | 0.12 | 30.79 | 4.04e-160 |
| **Number of loci** | **0.0038** | **0.00030** | **12.60** | **1.33e-34** |
| **Number of populations (K)** | **0.075** | **0.0074** | **10.16** | **1.91e-23** |
| Dispersal model_stepping stones | -0.095 | 0.11 | -0.86 | 0.39 |
| **Fst** | **3.77** | **0.16** | **23.27** | **7.79e-102** |
| Method_snapclust | 0.014 | 0.11 | 0.13 | 0.90 |
| Method_BAPS | 0.33 | 0.11 | 3.02 | 0.0025 |
| **Method_find.clusters** | **-0.56** | **0.11** | **-5.14** | **3.08e-07** |
| **Number of loci : Fst** | **-0.0059** | **0.00060** | **-9.08** | **3.45e-19** |
| Stepping stones : Method_snapclust | -0.0026 | 0.15 | -0.017 | 0.99 |
| Stepping stones : Method_BAPS | -0.048 | 0.15 | -0.31 | 0.75 |
| Stepping stones : Method_find.clusters | -0.49 | 0.15 | -3.16 | 0.0016 |

**Table S4: effect of different variables on the simulation results in the presence of hybrids, on the probability of correct assignment.** This table provides the summary of the final multivariate regression carried out on logit(*p*), where ‘*p*’ is the probability of correct group assignment. Bold font indicates significant results after Bonferroni correction with a target type 1 error of 1%. Contrasts were set for ‘Method’ with NEWHYBRIDS as the intercept.

|  | **Estimate** | **Std. Error** | **t value** | **Pr(>\|t\|)** |
| --- | --- | --- | --- | --- |
| Intercept | 0.65 | 0.17 | 3.85 | 0.00012 |
| **Number of loci** | **0.011** | **0.00050** | **23.32** | **2.74e-110** |
| Dispersal model | 0.12 | 0.045 | 2.73 | 0.0063 |
| **Fst** | **7.96** | **0.25** | **31.28** | **5.094e-185** |
| **Hybrid Class F1** | **-1.26** | **0.18** | **-7.18** | **8.56e-13** |
| **Hybrid Class BC1** | **-4.19** | **0.18** | **-23.87** | **4.82e-115** |
| **Hybrid Class BC2** | **-5.71** | **0.18** | **-32.58** | **2.077e-198** |
| **Method_snapclust** | **-1.43** | **0.18** | **-8.13** | **6.32e-16** |
| **Number of loci : Fst** | **-0.0052** | **0.0010** | **-5.046** | **4.78e-07** |
| **Hybrid Class F1 : Method_snapclust** | **1.63** | **0.25** | **6.55** | **6.59e-11** |
| **Hybrid Class BC1 : Method_snapclust** | **1.98** | **0.25** | **7.99** | **2.014e-15** |
| **Hybrid Class BC2 : Method_snapclust** | **2.99** | **0.25** | **12.07** | **9.019e-33** |

**Table S5: effect of different variables on the simulation results in the presence of hybrids, on the support to the real class.** This table provides the summary of the final multivariate regression carried out on logit(*p*), where ‘*p*’ is the support to the true group. Bold font indicates significant results after Bonferroni correction with a target type 1 error of 1%. Contrasts were set for ‘Method’ with NEWHYBRIDS as the intercept.

|  | **Estimate** | **Std. Error** | **t value** | **Pr(>\|t\|)** |
| --- | --- | --- | --- | --- |
| Intercept | 0.40 | 0.10 | 3.94 | 8.26e-05 |
| **Number of loci** | **0.0077** | **0.00030** | **26.51** | **9.85e-139** |
| **Dispersal model** | **0.11** | **0.027** | **4.082** | **4.60e-05** |
| **Fst** | **5.61** | **0.15** | **36.50** | **6.71e-240** |
| **Hybrid Class F1** | **-0.64** | **0.11** | **-5.99** | **2.29e-09** |
| **Hybrid Class BC1** | **-3.033** | **0.11** | **-28.62** | **1.023e-158** |
| **Hybrid Class BC2** | **-3.81** | **0.11** | **-36.00** | **2.14e-234** |
| **Method_snapclust** | **-1.34** | **0.11** | **-12.69** | **6.27e-36** |
| **Number of loci : Fst** | **0.0033** | **0.00060** | **5.37** | **8.72e-08** |
| **Hybrid Class F1 : Method_snapclust** | **0.69** | **0.15** | **4.61** | **4.20e-06** |
| **Hybrid Class BC1 : Method_snapclust** | **1.24** | **0.15** | **8.30** | **1.56e-16** |
| **Hybrid Class BC2 : Method_snapclust** | **1.90** | **0.15** | **12.67** | **7.96e-36** |


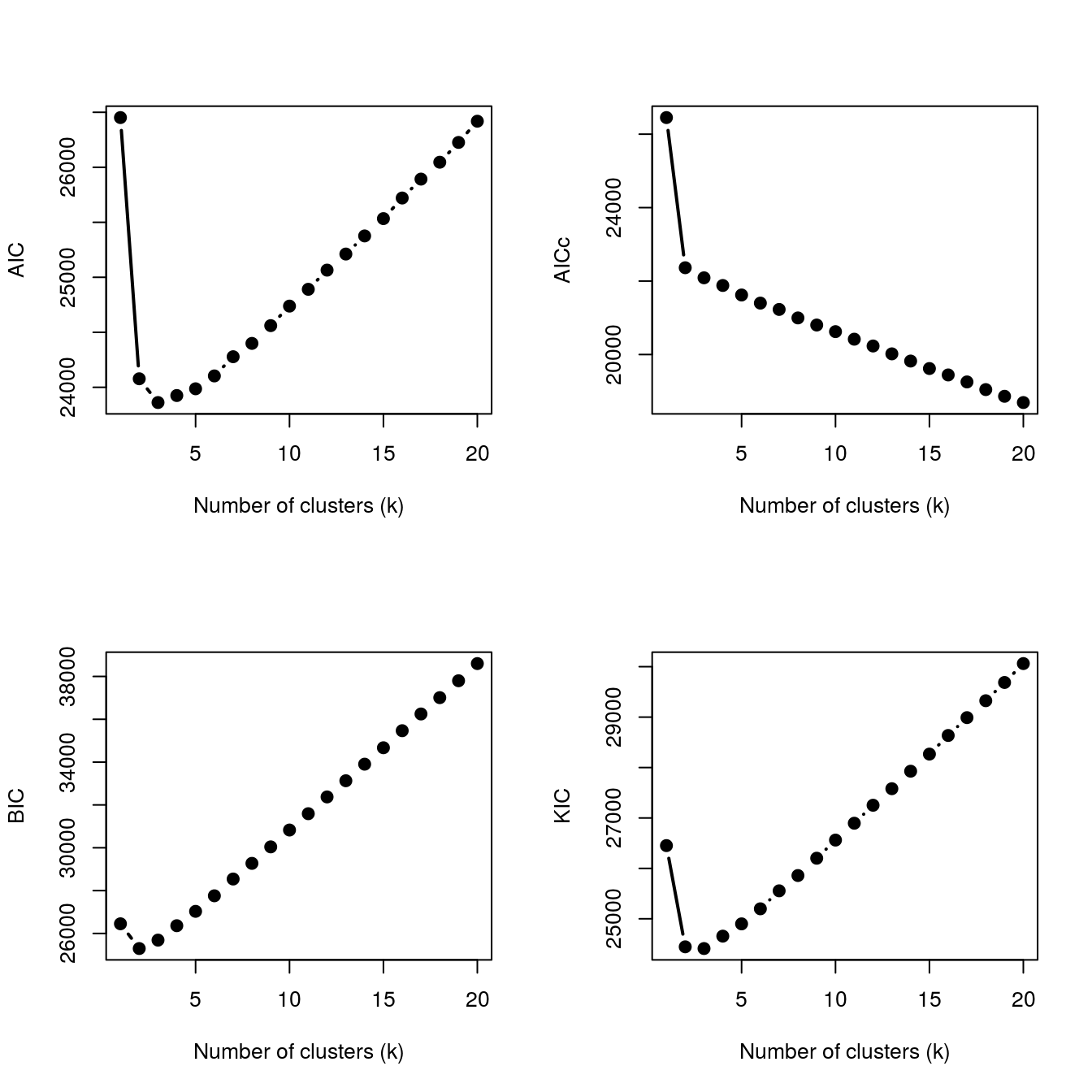


**Figure S1: selection of the optimal number of clusters for the microbov example.** This figure shows results of the 4 goodness-of-fit statistics implemented for *snapclust* (AIC, AICc, BIC, and KIC), identifying 2 optimal clusters in from the microbov example dataset.


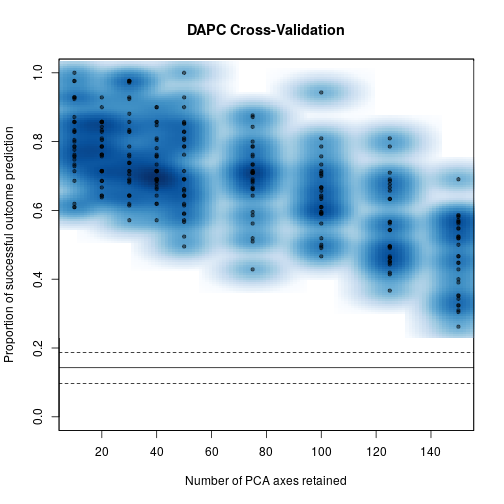


**Figure S2: Output of the cross-validation analysis carried out to determine the number of principal components to retain in the DAPC led on the microbov example.** This figure shows the proportion of successful group re-assignment via cross-validation for varying numbers of PCA axes retained, using 90% of individuals for the training set. For each number of PCA axes, 30 independent replicates were obtained. The density of observation is indicated in blue shades. The plain and dashed lines indicate the mean expectation from a random classifier, and its 95% confidence interval.
